# Prostate Cancer-Specific and Potent Antitumor Effect of a *DD3*-Controlled Oncolytic Virus Harboring the *PTEN* Gene

**DOI:** 10.1371/journal.pone.0035153

**Published:** 2012-04-11

**Authors:** Miao Ding, Xin Cao, Hai-neng Xu, Jun-kai Fan, Hong-ling Huang, Dong-qin Yang, Yu-hua Li, Jian Wang, Runsheng Li, Xin-Yuan Liu

**Affiliations:** 1 State Key Laboratory of Cell Biology, Institute of Biochemistry and Cell Biology, Shanghai Institutes for Biological Sciences, Chinese Academy of Sciences, Shanghai, China; 2 Xinyuan Institute of Medicine and Biotechnology, College of Biological Sciences, Zhejiang Sci-Tech University, Hangzhou, China; 3 Department of Molecular and Cellular Biochemistry, Markey Cancer Center, University of Kentucky, Lexington, Kentucky, United States of America; 4 Key Laboratory of Contraceptive Drugs and Devices of NPFPC, Shanghai Institute of Planned Parenthood Research, Shanghai, China; University of Chicago, United States of America

## Abstract

Prostate cancer is a major health problem for men in Western societies. Here we report a Prostate Cancer-Specific Targeting Gene-Viro-Therapy (CTGVT-PCa), in which PTEN was inserted into a DD3-controlled oncolytic viral vector (OV) to form Ad.DD3.E1A.E1B(Δ55)-(PTEN) or, briefly, Ad.DD3.D55-PTEN. The woodchuck post-transcriptional element (WPRE) was also introduced at the downstream of the E1A coding sequence, resulting in much higher expression of the E1A gene. DD3 is one of the most prostate cancer-specific genes and has been used as a clinical bio-diagnostic marker. PTEN is frequently inactivated in primary prostate cancers, which is crucial for prostate cancer progression. Therefore, the Ad.DD3.D55-PTEN has prostate cancer specific and potent antitumor effect. The tumor growth rate was almost completely inhibited with the final tumor volume after Ad.DD3.D55-PTEN treatment less than the initial volume at the beginning of Ad.DD3.D55-PTEN treatment, which shows the powerful antitumor effect of Ad.DD3.D55-PTEN on prostate cancer tumor growth. The CTGVT-PCa construct reported here killed all of the prostate cancer cell lines tested, such as DU145, 22RV1 and CL1, but had a reduced or no killing effect on all the non-prostate cancer cell lines tested. The mechanism of action of Ad.DD3.D55-PTEN was due to the induction of apoptosis, as detected by TUNEL assays and flow cytometry. The apoptosis was mediated by mitochondria-dependent and -independent pathways, as determined by caspase assays and mitochondrial membrane potential.

## Introduction

Prostate cancer is a major health problem for men in Western societies. Each year, approximately 230,000 American males are diagnosed with prostate cancer and nearly 30,000 die from this disease [1], [2]. The best available treatment for patients with the advanced disease is androgen ablation therapy, based on the observations of Huggins and Hodges [3] that clinical prostate cancer is under the trophic influence of male hormones. Tumor regression and improvement of clinical symptoms are temporary and the disease inevitably progresses to an androgen-independent state. Currently, no curative therapy is available for androgen-independent prostate cancers.

Bio-therapy provides an attractive opportunity to target androgen-independent prostate cancers. Unlike traditional chemotherapy, it can be designed and customized to specifically target cancers according to our understanding of the disease at a molecular level. The adenoviral vector has been used as a transfer vehicle to introduce genes into cancer cells because it is more efficient than non-viral gene transfer methods [4], [5]. The adenoviral vector is stable *in vivo*, efficiently delivers genes to both dividing and non-dividing cells and rarely causes any significant disease itself [6], [7]. However, the traditional adenovirus used for gene therapy is a replication deficient one with low expression level of the therapeutic gene. Cancer-specific replication of the oncolytic vector, therefore, is absolutely required to prevent these problems. Two major strategies have been used to construct a replicative and cancer-specific oncolytic adenovirus. The first is to delete the viral element that is necessary for virus replication in normal cells, which is not required in tumor cells. For example, the E1B-55K gene, which was deleted in the oncolytic viruses ONYX-015 and ZD55 [8], [9], [10], is required for viral replication in normal cells but is dispensable in cancer cells due to compensatory mechanisms. The second strategy is to replace the promoter of a key gene in adenovirus replication, such as the E1A or E1B gene, with a tumor -specific promoter [11], [12]. Thus, the oncolytic virus is highly replicated when the promoter is activated.

To overcome the limitations of traditional gene therapy with an replication deficient adenoviral vector, a Cancer-Targeting Gene-Viro-Therapy (CTGVT) was constructed by inserting an anti-tumor gene into a double-targeted oncolytic viral(OV) vector. It is actually an OV-gene, and has much better antitumor effect than that of either gene therapy alone or virotherapy alone. The oncolytic virus itself has anti-tumor power and selectively replicates several hundred-fold in tumor cells and thus, the therapeutic genes should also be selectively replicated several hundred-fold in tumor cells [11]. By innovatively integrating gene therapy and viro-therapy, the anti-tumor effect of the CTGVT is generally much higher than either method alone. On January 27, 2011, Amgen spent 1 billion USD to purchase OncoHSV-GM-CSF (an oncolytic virus from Herpes Simplex Virus-1), which is in phase III for advanced melanoma [13], [14], underlining the importance and potential of the CTGVT strategy.

In this study, we first generated a double-regulated adenovirus, Ad.DD3.D55, in which the E1A gene is under the control of *DD3* promoter and the E1B-55K gene was deleted. *DD3* is one of the most prostate cancer-specific genes and has been used as a clinical biomarker for prostate cancer [15]. Interestingly, a 214-bp fragment of the DD3 core promoter has a high promoter activity [16], and therefore it was used to control E1A expression in the oncolytic adenovirus. Because DD3 expression is highly restricted in prostate cancer, we previously used the minimal DD3 promoter to drive expression of E1A for generating an oncolytic virus [17]. The level of DD3 promoter-driven E1A expression was insufficient, therefore, WPRE was introduced to enhance the expression of the E1A gene in this study. WPRE promotes gene expression in a promoter- and cell-line-dependent manner [18]. WPRE has been exploited as an enhancer of transgene expression by enhancing nuclear export of an aberrantly retained messenger RNA from the nucleus to the cytoplasm [19]. In addition, *PTEN* is a well-known tumor suppressor gene that encodes a protein phosphatase [20]. Its deletion has been observed in high-grade prostate cancer and is very important for prostate cancer progression [21]. In this study, *PTEN* with a CMV promoter was inserted into Ad.DD3.D55 to form Ad.DD3.D55-PTEN, which has a prostate cancer replication specificity and antitumor gene specificity. Ad.DD3.D55-PTEN efficaciously induces apoptosis in prostate cancer cells, eliminating prostate cancer xenografts with higher antitumor efficiency.

## Results

### Prostate Cancer-Specific Transcriptional Activity of the DD3 Promoter and cytopathic effect of Ad.DD3.D55

**Figure 1:**
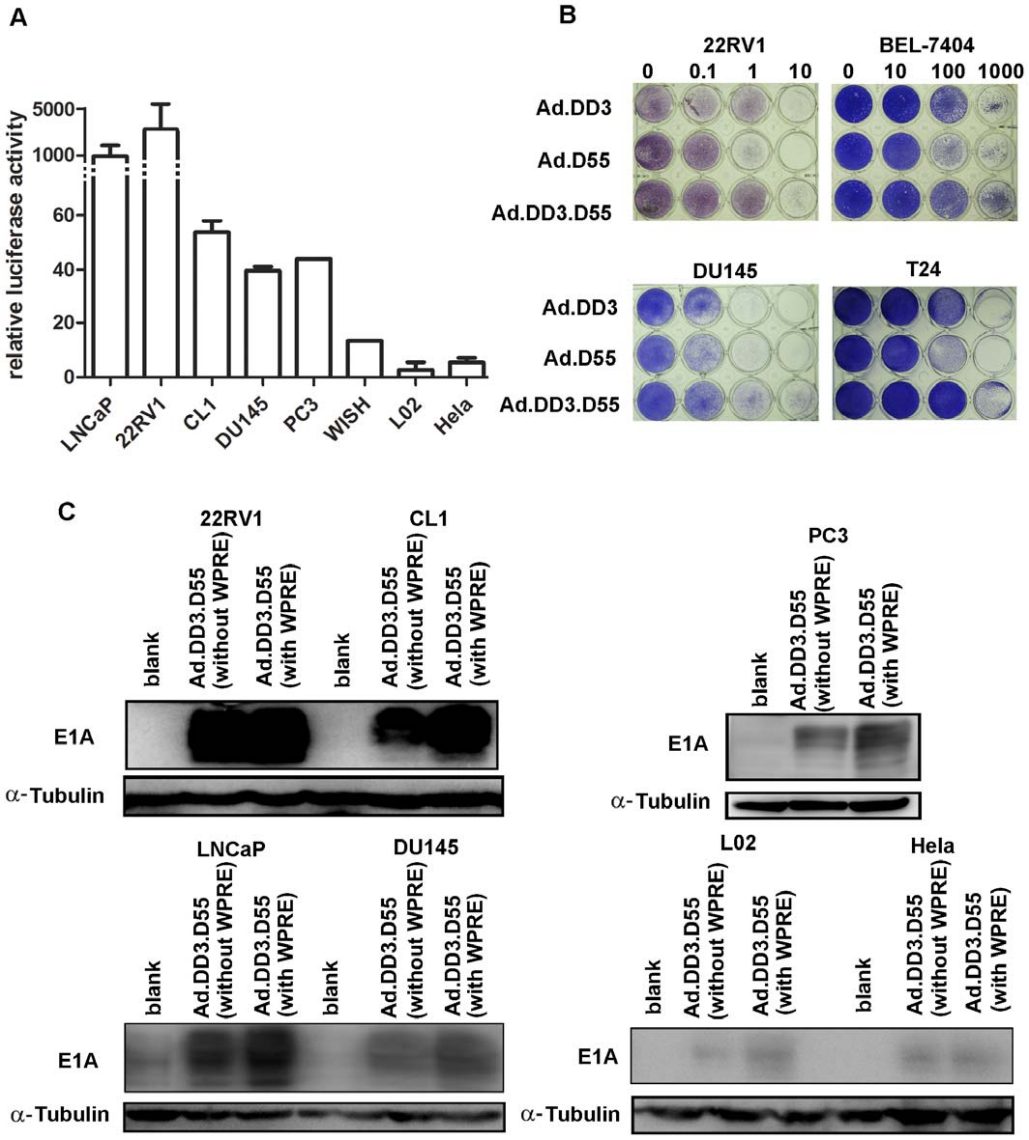
Prostate cancer-specific expression driven by the *DD3* promoter in the presence of WPRE. A. The luciferase activity of the DD3 promoter in the presence of WPRE is expressed compared to the pGL3-control (activity = 1000). Cells were seeded in 24-well plates. Forty-eight hours after infection with the reporter plasmids pGL3-control or pGL3-DD3-WPRE, the cells were harvested, and the luciferase activity was measured. The activity of pGL3-DD3-WPRE relative to the pGL3-control is presented as the mean ± standard deviation (SD) of three transfections. B. The expression of E1A by Ad.DD3.D55 (without WPRE) and Ad.DD3.D55 in different cell lines was evaluated. α-Tubulin was evaluated as a loading control. C. Crystal violet assay. Cells on 24-well plates were infected with different viruses at the indicated MOIs. Cytotoxicity was observed by crystal violet staining at five days after infection.

The minimal DD3 promoter activity in the absence of WPRE was measured by luciferase-reporter expression, which showed the minimal DD3 promoter typically exhibited much more activity in the prostate cancer cell lines than in other cell lines. The pGL3-control, which contains the SV40 promoter and enhancer, was used as a positive control (activity = 1000). As shown in Figure 1A, expression driven by the *DD3* promoter with WPRE was much higher in LNCaP and 22RV1 than in other cells, including the prostate cancer cell lines DU-145,PC3 and CL1. Although there were differences in the levels of expression in these prostate cell lines, these results indicate that WPRE could mediate enhancement of gene expression and maintain the relatively restricted activity of the DD3 promoter in prostate cell lines.

**Figure 2:**
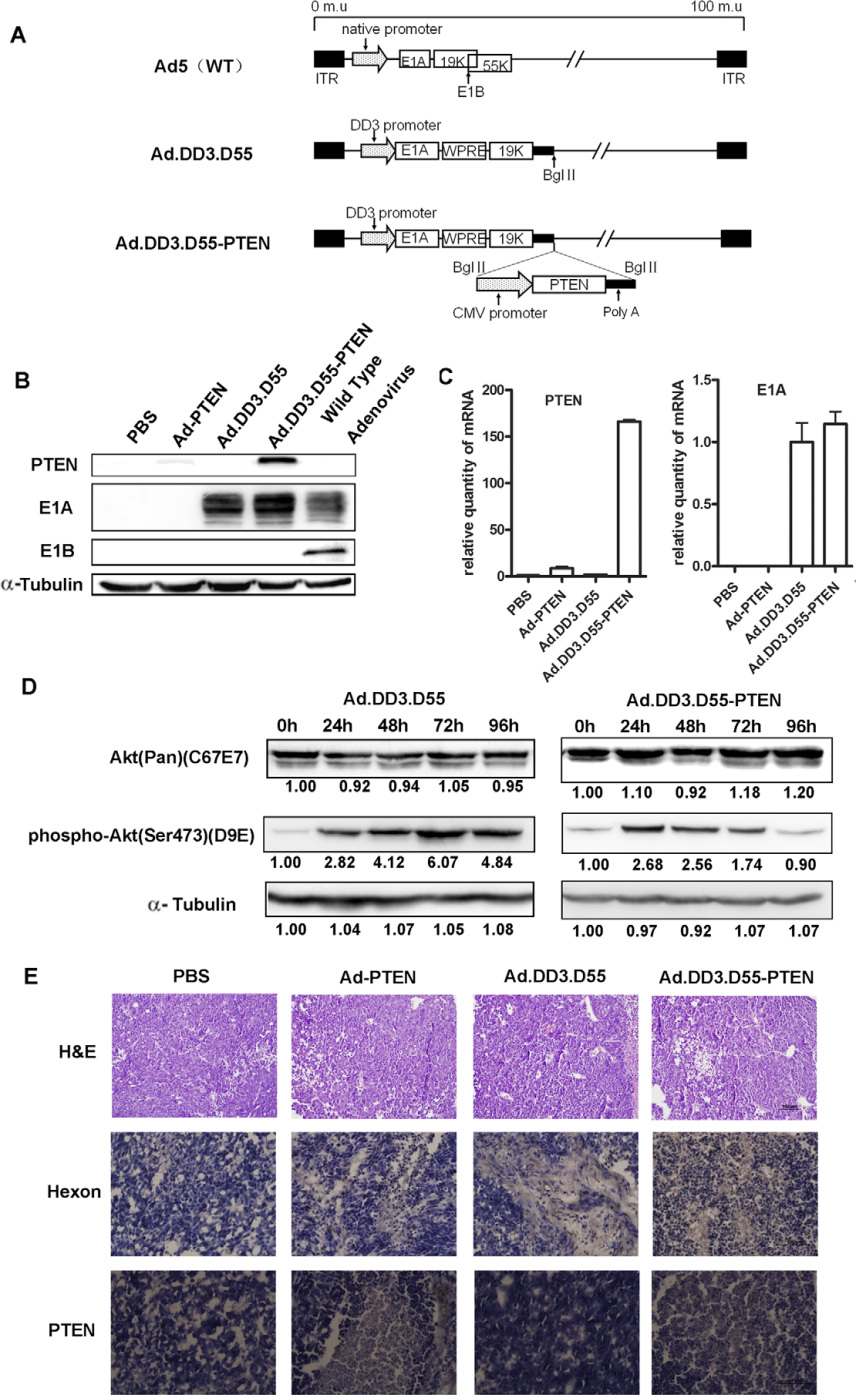
Characterization of Ad.DD3.D55-PTEN. A. Schematic diagram of Ad.DD3.D55-PTEN. The endogenous promoter of E1A was replaced by the DD3 promoter, and the E1B-55K gene was deleted. The *PTEN* expression cassette was inserted into Ad.DD3.D55 to construct Ad.DD3.D55-PTEN. ITR, inverted terminal repeat. B. Characterization of Ad-PTEN, Ad.DD3.D55 and Ad.DD3.D55-PTEN expression by Western blot analysis. The expression of PTEN, E1A and E1B-55K by Ad-PTEN, Ad.DD3.D55 or Ad.DD3.D55-PTEN in CL1 cells was evaluated. Ad.WT was used as a positive control. α-Tubulin was evaluated as a loading control. C. Relative mRNA levels of *PTEN* and E1A in 22RV1 cells infected with Ad-PTEN, Ad.DD3.D55 and Ad.DD3.D55-PTEN. GAPDH was evaluated as an internal reference. The data are presented as the mean ± SD of three separate experiments. D. Western blot analysis of the total and phosphorylated Akt protein levels in CL1 cells infected with Ad.DD3.D55 and Ad.DD3.D55-PTEN. E. Analysis of tumor sections derived from tumors treated with PBS or one of various adenoviruses by hematoxylin and eosin staining and hexon and PTEN immunostaining. Scale bar, 100 µm.

We constructed a new oncolytic virus, Ad.DD3-E1A.E1B(Δ55), briefly Ad.DD3.D55 (Fig. 2A), which is a double-regulated adenovirus in which the native promoter of E1A has been replaced by the minimal *DD3* promoter and the E1B-55K gene has been deleted. The cytopathic effect of Ad.DD3.D55 was evaluated in normal and tumor cells by crystal violet staining(Fig. 1B). In prostate cancer cell lines 22RV1 and DU145, the cytoxicity of Ad.DD3.D55 was comparable to the single-regulated adenovirus Ad.DD3 and Ad.D55. In contrast, in the non prostate cancer cell BEL-7404 and T24, the cytopathic effect of Ad.DD3.D55 and Ad.DD3 was much lower, indicating that the prostate cancer-specificity of cytoxicity exerted by Ad.DD3.D55 was much more improved.

Then we measured expression levels of E1A in the different cells infected with Ad.DD3.D55 (with WPRE) or Ad.DD3.D55(without WPRE) (Fig. 1C), respectively. Expression of E1A was strongest in the 22RV1 cells among all the cell lines tested, and did not change significantly in the presence of WPRE. However, introduction of WPRE indeed raised the expression levels of E1A in several prostate cancer cell line including CL1, LNCaP, PC3 and DU-145. Besides it, the data demonstrated that introduction of WPRE did not significantly affect the restricted expression of E1A driven by DD3 promoter in prostate cancer cells, providing a strong piece of evidence supporting that WPRE is a promising candidate for constructing an oncolytic adenovirus to target prostate cancer cells.

### Construction and Characterization of Ad.DD3.D55-PTEN

The *PTEN* expression cassette was introduced into the DD3 controlling double regulated-adenovirus Ad.DD3.D55 to generate Ad.DD3.D55-PTEN (Fig. 2.A). The expression levels of PTEN and E1A in different CTGVT viruses-infected cells were determined by Western blotting after infecting the human prostate cancer cell line CL1 (Fig. 2B). The replication-deficient adenovirus Ad-PTEN with E1 region deletion and the double-regulated adenovirus Ad.DD3.D55 were used as controls. As expected, the double-regulated viruses Ad.DD3.D55 and Ad.DD3.D55-PTEN expressed E1A protein at approximately the same levels as did Ad.WT and failed to express the E1B-55K protein. Similar results were obtained when protein expression was measured at the mRNA level by real-time PCR (Fig. 2C). To characterize the function of PTEN in Ad.DD3.D55-PTEN, the effects of its expression on the phosphorylation status of Akt were examined by Western blotting. As shown in Fig. 2D, infection by the oncolytic virus led to an increase in phospho-Akt expression which was detectable as early as 24 h after infection. Additionally, PTEN expression inhibited phosphorylation of Akt at its activating residue (Ser473) without diminishing total Akt protein levels compared to the vector Ad.DD3.D55. These results are consistent with PTEN's lipid phosphatase activity. To further characterize the viruses *in vivo*, levels of the viral protein hexon and the therapeutic gene *PTEN* were measured in tumor specimens 7 days after injection of the viruses. As shown in Figure 2E, high levels of hexon were observed in the Ad.DD3.D55 and Ad.DD3.D55-PTEN groups. The expression of the therapeutic gene *PTEN* was also measured in these tumor specimens. The samples infected with Ad-PTEN and Ad.DD3.D55-PTEN displayed high PTEN expression levels, but *PTEN* expression was not observed in the Ad.DD3.D55-treated group.

### Prostate Cancer-Specific Cytotoxicity of Ad.DD3.D55-PTEN *In Vitro*

**Figure 3:**
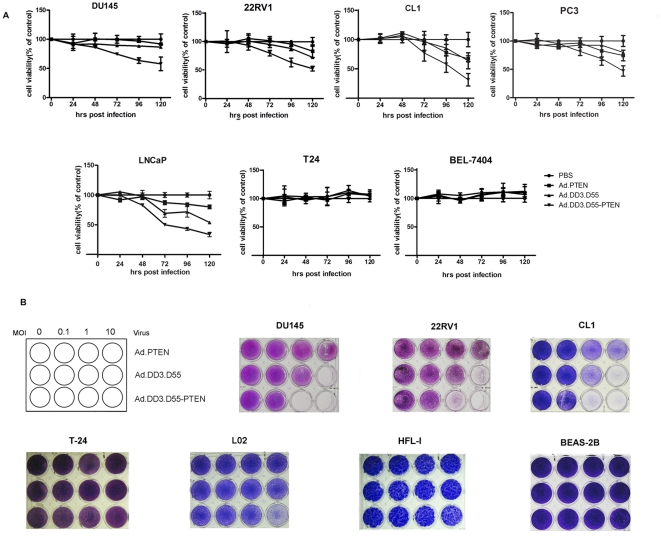
Prostate cancer-specific cytotoxicity of Ad.DD3.D55-PTEN. A. MTT assay. The six cell lines indicated above were seeded on 96-well plates and infected with different viruses at an MOI of 10. Cell viability was measured by MTT assays at 0, 24, 48, 72, 96 and 120 hours post-infection. The results are presented as the mean ± SD of five separate experiments and are expressed as the percentage relative to mock-treated control cells. B. Crystal violet assay. Cells on 24-well plates were infected with different viruses at the indicated MOIs. Cytotoxicity was observed by crystal violet staining at five days after infection.

The cytotoxicity of Ad.DD3.D55-PTEN was evaluated by MTT assay in three prostate cancer cell lines CL1, LNCaP, DU145, 22RV1, PC3 and two non-prostate cancer cell lines T24(human urinary bladder carcinoma cell line) and BEL-7404(human liver cancer cell line). The results showed that growth of infected prostate cancer cells were much inhibited with different degree, while the highest efficiency of inhibition by Ad.DD3.D55-PTEN was observed in LNCaP cells and CL1 cells (Fig. 3A). These data also showed that cytotoxicity of Ad.DD3.D55-PTEN is significantly higher than that of Ad.DD3.D55. However, both of virus were shown to be unable to inhibit growth of non-prostate cancer cells significantly (Fig. 3A).

The cytotoxicity of Ad.DD3.D55-PTEN was also analyzed by staining with crystal violet as shown in Figure 3B. Ad.DD3.D55-PTEN had a strong cytotoxic effect on the prostate cancer cells. LNCaP cells and CL1 cells were more sensitive to the cytotoxicity exerted by Ad.DD3.D55-PTEN than were DU-145 and 22RV1 cells. However, little or no cytotoxicity was observed in non-prostate cancer cell lines, such as T-24, BEL-7404, and normal cell lines, HFL-I and BEAS-2B. These results indicate that the cytotoxicity of Ad.DD3.D55-PTEN was not only much stronger than that of Ad.DD3.D55 due to the expression of *PTEN*, but was also highly restricted to prostate cancer cells.

### Ad.DD3.D55-PTEN Induces Apoptosis in Prostate Cancer Cells *In Vitro*

**Figure 4:**
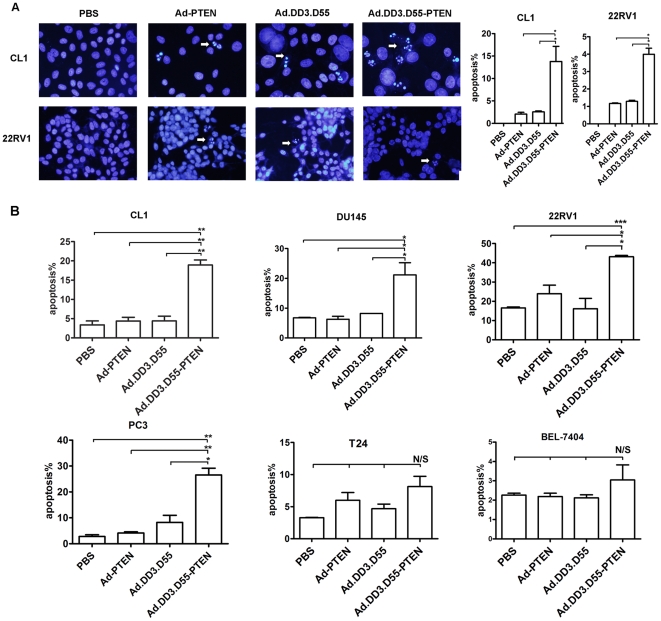
Ad.DD3.D55-PTEN induces apoptosis of prostate cancer cells. A. CL1 and 22RV1 cells were stained with Hoechst 33258 at 48 hours after viral infection. The arrows indicate the apoptotic cells. Scale bar, 10 µm. B. Flow cytometric analysis of propidium iodide-stained cells was performed at 48 hours after infection. The percentages of sub-G1 cells are shown. The results are presented as the mean ± SD of three separate experiments (** indicates *P*<0.01; * indicates *P*<0.05; N/S indicates *P*>0.05).

We stained cells with Hoechst33258 to further analyze the apoptosis-inducing capacity of Ad.DD3.D55-PTEN *in vitro*. As shown in Figure 4A, Ad.DD3.D55-PTEN induced significant apoptosis in CL1 and 22RV1 cells. However, replication-incompetent Ad-PTEN and double-regulated Ad.DD3.D55 exhibited a marginal ability to induce apoptosis. The flow cytometry was performed in which dead cells were represented by the fraction of cells in the sub-G1 phase. Infection with Ad.DD3.D55-PTEN increased the number of cells in the sub-G1 phase in the tested prostate cell lines(CL1, DU145, 22RV1, PC3) and a higher apoptotic index was found in 22RV1 cells (43.135%) than in DU145, PC3 or CL1 cells, consistent with the higher expression of E1A in the 22RV1 cell line(Fig. 4B).

### Ad.DD3.D55-PTEN Induces Apoptosis through Intrinsic and Extrinsic Signaling Pathways

**Figure 5:**
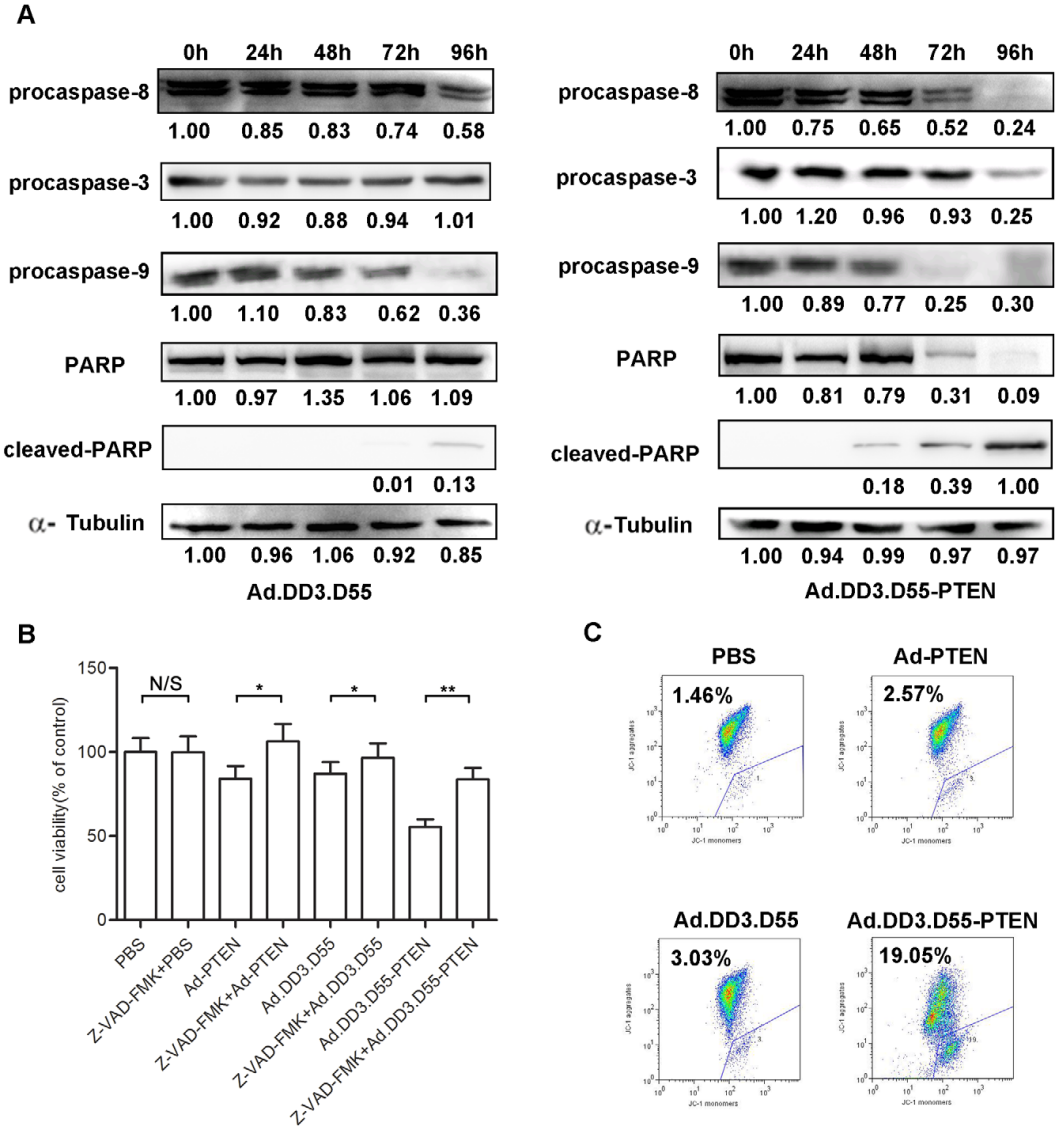
Ad.DD3.D55-PTEN activates caspase-mediated apoptosis signaling pathways. A. Western blot analysis of apoptosis-related proteins in CL1 cells infected with Ad.DD3.D55 or Ad.DD3.D55-PTEN. B. CL1 cells were treated with the indicated agents and cell viability was analyzed by the MTT assay after 120 hours. For the Z-VAD-fmk inhibition assay, cells were pre-treated with Z-VAD-fmk (20 µM) 2 h before virus infection. The results are presented as the mean ± SD of three separate experiments (** indicates *P*<0.01; * indicates *P*<0.05; N/S indicates *P*>0.05). C. Analysis of the mitochondrial membrane potential of CL1 cells by FACS after JC-1 staining. The percentage of cells in the trapeziform region is shown.

The mechanism of Ad.DD3.D55-PTEN-induced apoptosis was studied. As shown in Figure 5B, the pan-caspase inhibitor Z-VAD-fmk inhibited the cell death induced by Ad.DD3.D55-PTEN in CL1 cells. This result indicates that the cell death induced by Ad.DD3.D55-PTEN is caspase-dependent. Additionally, a series of caspase-dependent apoptosis signaling cascades were analyzed by Western blotting (Fig. 5A). Enhanced PARP cleavage and activation of procaspase-3, procaspase-8 were detected in Ad.DD3.D55-PTEN-infected cells (Fig. 5A). We also detected a time-dependent reduction in level of precaspase-9 in Ad.DD3.D55-PTEN-infected cells (Fig. 5A), consistent with the activation of the mitochondria-mediated apoptosis signaling pathway. The integrity of the cell mitochondria was evaluated with the potentiometric fluorescent dye JC-1(Fig. 5B). Flow cytometry revealed that infection with Ad.DD3.D55-PTEN resulted in a significant decrease in mitochondrial transmembrane electrical potential (Fig. 5C). Taken together, these observations indicate that PTEN effectively enhanced Ad.DD3.D55-induced apoptosis by intrinsic and extrinsic pathways.

### Suppression of Tumor Growth by Ad.DD3.D55-PTEN Induces Apoptosis *In Vivo*

**Figure 6:**
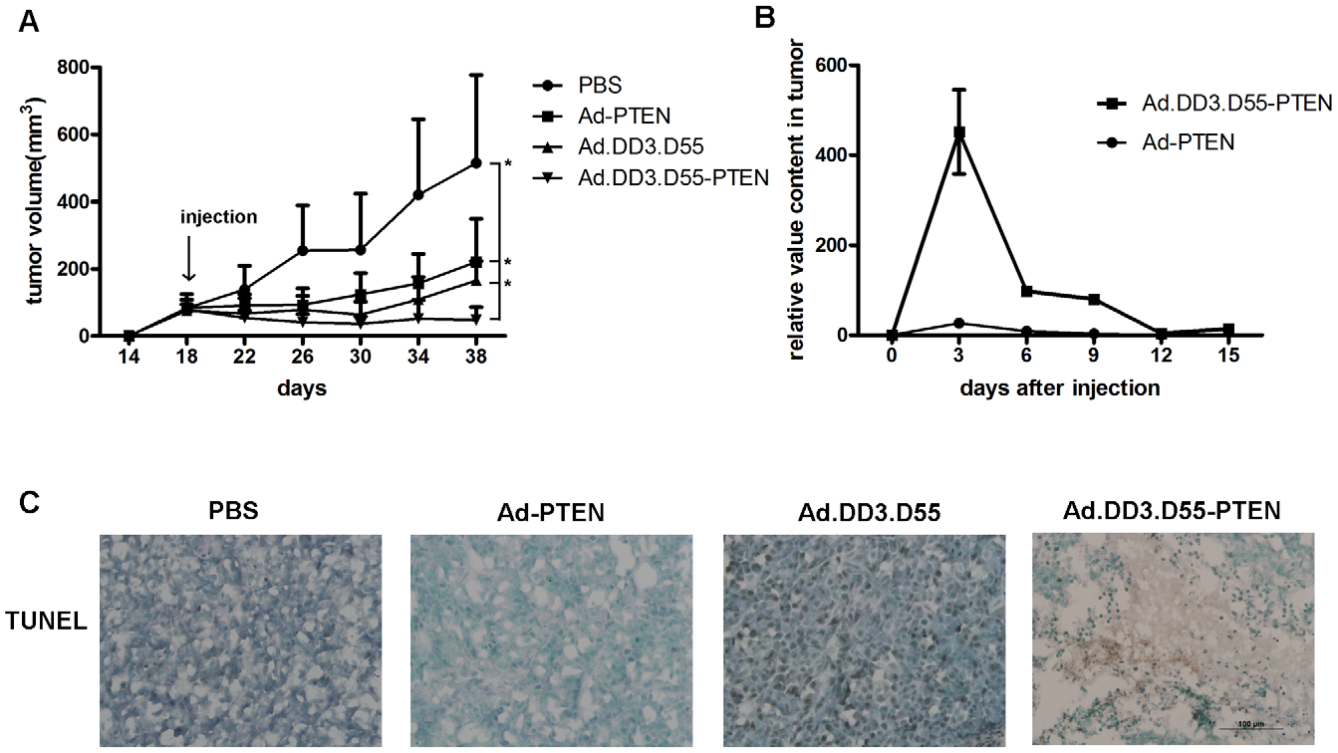
Ad.DD3.D55-PTEN inhibits the growth of prostate cancer cell xenograft tumors. A. Antitumor effect of infection of CL1 xenograft tumors with different adenoviruses. The tumor volume was measured and is presented as the mean ± SD. B. Ad.DD3.D55-PTEN inhibits the growth of CL1 xenograft tumors. Analysis of the viral kinetics of Ad-PTEN or Ad.DD3.D55-PTEN-treated 22RV1 tumors by real-time PCR. Adenovirus E3 region was amplified to evaluate the viral DNA content. β-actin DNA was used as an internal control. (NS means *P*>0.05, no statistical significance). C. Analysis of tumor sections derived from tumors treated with PBS or one of various adenoviruses by TUNEL staining. Scale bar, 100 µm.

The results presented above show that the anti-tumor effect of the CTGVT agent Ad.DD3.D55-PTEN is much better than either the oncolytic virus Ad.DD3.D55 or Ad.PTEN alone. Of the prostate cancer cell lines tested, Ad.DD3.D55-PTEN killed LNCaP and CL-1 cells with the highest efficiency. We selected the cell line CL1, an androgen-independent subclone of LNCaP, to evaluate the oncolytic potential of Ad.DD3.D55-PTEN because LNCaP cells have poor tumor-forming capability *in vivo*. As shown in Figure 6A, the tumor growth after injection of was almost completedly inhibited with the final tumor volume after Ad.DD3.D55-PTEN treatment less than the initial volume at the beginning of Ad.DD3.D55-PTEN treatment. Another way of saying, not only did Ad.DD3.D55-PTEN-infected tumors not grow, but their volumes actually shrunk by 41.3% when measured 20 days after the injection. This shows the powerful effect of Ad.DD3.D55-PTEN on tumor growth. However, both Ad.DD3.D55 and Ad.PTEN inhibited tumor growth with a much lower efficiency, compared to that of Ad.DD3.D55-PTEN.

The viral kinetics study *in vivo* further illustrate the superiority of Ad.DD3.D55-PTEN with its viral DNA quantity up to 100 times greater than that of Ad-PTEN within 72 h and lasting for 2 weeks (Fig. 6B). TUNEL analysis revealed that injection of Ad.DD3.D55-PTEN caused significant amount of apoptosis in the tumors, while no apoptosis was observed in PBS-treated tumors, and little in Ad-PTEN-treated tumors(Fig. 6C).

## Discussion

In this study, Ad.DD3.D55-PTEN demonstrated an excellent antitumor effect *in vitro* and *in vivo*. Two major factors contribute to the cytotoxicity exerted by Ad.DD3.D55-PTEN. The first is that it replicates selectively in prostate cancer cells. We previously reported that the minimal DD3 promoter-controlled oncolytic virus replicated selectively in prostate cancer cells [17], although the promoter activity was not very satisfactory. PSA is a well-known biomarker of prostate cancer and its promoter has been widely used to study biotherapies for prostate cancer, including gene therapy with different vectors [22], [23], [24], [25]. Different recombinant promoters have been generated based on the Prostate Cancer-specific activity of a promoter or enhancer to increase the Prostate Cancer-specific activity of the promoter controlling the replication of the oncolytic virus [26], [27], [28], [29]. Cell killing efficacy could be improved by many methods, such as combining the oncolytic adenovirus with radiotherapy or chemotherapy. Clinical trials have shown that the combination of ONYX-15 virus and chemotherapy elicits a stronger anti-tumor response against head and neck cancer than ONYX-15 or chemotherapy alone [30]. Patients with prostate cancer have been treated most frequently with androgen ablation. Because the activity of the PSA promoter/enhancer is highly dependent on endogenous androgen [31], the *in vivo* cytotoxicity of a PSA promoter/enhancer-based oncolytic virus could be reduced significantly if it was used in patients who have been treated with androgen ablation. In this study, we introduced WPRE in the DD3-controlled E1A expression cassette. Interestingly, the presence of WPRE raised the levels of reporter expression considerably but did not significantly affect the restricted expression in prostate cancer cells. To our knowledge, this is the first reported use of WPRE in the generation of an oncolytic virus. In addition, our data show that Ad.DD3.D55-PTEN, in which E1A expression is under the control of the DD3 promoter and WPRE, exerted a strong inhibitory effect on the growth of androgen-dependent as well as androgen-independent cell lines, including CL1, DU145 and 22RV1 in cell culture and CL1 tumor growth in a xenograft nude mouse. Ad.DD3.D55-PTEN has a powerful cytotoxic effect in androgen-independent cell lines. Therefore, E1A expression under the control of the DD3 promoter and WPRE may be clinically significant for oncolytic viral therapy of patients with prostate cancer.

Our results showed that the expression of PTEN played the second key role in the cytotoxicity of Ad.DD3.D55-PTEN. PTEN acts as a phosphatidylinositol phosphatase with a possible role in phosphatidylinositol 3′-kinase (PI3′K)-mediated signal transduction, suppressing the PI3K/AKT pathway by reducing the level of PI3′K in the cell [32], [33]. The oncolytic virus Ad.DD3.D55 and Ad.DD3.D55-PTEN led to an increase in phospho-Akt expression at 24 h after infection(Fig. 2.D); the activation of PI3K-Akt signaling is the pathway employed by viruses for viral endocytosis and replication [34], [35], [36], [37]. Ad.DD3.D55-PTEN inhibited phosphorylation of Akt at its activating residue (Ser473) as a result of PTEN expression, while the activation of Akt was maintained with the infection of Ad.DD3.D55(Fig. 2.D). The difference clearly indicated the powerful role of PTEN. In addition, PTEN has been identified as the inactivating alteration in multiple human cancer types, such as glioma, breast, lung, prostate, bladder, melanoma and kidney tumors, astrocytoma, and leukemia [38]. In prostate cancer, the loss of the PTEN protein is correlated with tumors of high grade and stage, suggesting that alterations in the *PTEN* gene may be associated with prostate cancer progression [21], [39]. Wu et al. established a mouse model with prostate-specific deletion of PTEN [40]. This mouse model recapitulates prostate cancer progression in humans: initiation of prostate cancer with prostatic intraepithelial neoplasia, followed by progression to invasive adenocarcinoma and subsequent metastasis. Therefore, PTEN is an optimal candidate for cancer gene therapy due to its unique properties. Introduction of PTEN by adenovirus or retrovirus has been studied for bio-therapy of various type of cancer, including ovarian, endometrial, bladder, gastric, colorectal, and esophageal cancers and glioblastoma [41], [42], [43], [44], [45], [46], [47]. For example, Gallick et al. reported that the adenoviral-mediated expression of PTEN inhibited proliferation and metastasis of human prostate cancer cells *in vitro* and in *vivo* [48]. Adenoviral-mediated PTEN transgene expression combined with radiotherapy, chemotherapy and other cancer-suppressing genes has been used to treat prostate cancer cells to boost the antitumor effect [49], [50], [51], [52]. In this study, high levels of PTEN expression driven by the CMV promoter were mediated by an oncolytic virus that replicated selectively in prostate cancer cells by using DD3 promoter to drive E1A expression. The antitumor efficacy of PTEN in the oncolytic virus was much enhanced compared with the adenovirus-mediated PTEN that was previously reported for use in prostate cancer [48], [52], it could be resulted from that Ad.DD3.D55-PTEN induced a massive apoptosis in prostate cancer cells by intrinsic and extrinsic pathways.

We observed varying sensitivities of the different prostate cancer cell lines to the cytotoxicity of Ad.DD3.D55-PTEN. The level of reporter expression driven by the DD3 promoter and WPRE in LNCaP cells was only 21.79% of that in 22RV1 cells (Fig. 1), suggesting a lower replication of Ad.DD3.D55-PTEN in LNCaP cells, which would seem inconsistent with the higher killing efficacy of Ad.DD3.D55-PTEN in LNCaP cells than in 22RV1 cells (Fig. 3). However, 22RV1 cells express a functional PTEN, while LNCaP cells do not [53]. Given the above data, Ad.DD3.D55-PTEN probably exerts a stronger cytotoxic effect in PTEN-negative PCa cells than in PTEN-positive PCa cells.

Volumes of tumor of CL1 cells was redued by 41.3% 20 days after infected with Ad.DD3.D55-PTEN in the nude mouse (Fig. 6A) strongly suggests that Ad.DD3.D55-PTEN has the therapeutic potential for the treatment of androgen-independent cancers. This study revealed that adenoviral replication slowed after approximately 12 days of active replication (Fig. 6B). Interestingly, even the rate of oncolytic virus kept very low *in vivo* after the period (Fig. 6B), the infected tumor still did not significantly grow(Fig. 6A). Its mechanism for this phenomenon remains unknown. One plausible explanation is that a vast majority of cancer cells was killed in the first days after injection of Ad.DD3.D55-PTEN, while growth of the remaining cancer cells is efficiently inhibited even in the presence of a minimal amount of oncolytic virus. The phenomenon is probably encouraging since most of virus will be cleared up *in vivo* when the oncolytic virus are used clinically.

In conclusion, we developed a novel prostate-specific CTGVT(CTGVT-PCa), Ad.DD3.D55-PTEN, by controlling the expression of the E1A gene with a minimal DD3 promoter and WPRE. Ad.DD3.D55-PTEN had excellent anti-tumor efficacy in both androgen-dependent and -independent prostate cancer cell lines. This report also provides a new strategy for constructing a CTGVT-PCa with tumor types specificity that have few tumor-specific promoters available.

## Materials and Methods

### Ethics Statement and Animal Experiment

Male BALB/c nude mice (4-week-old) were maintained and used in a light and temperature controlled room in an AAALAC-accredited facility, and given water and lab chow *ad libitum* [17]. All experimental procedures were approved by the Institutional Animal Care and Use Committee of Shanghai Institute of Biochemistry and Cell Biology under protocol IBCB-SPF0029. To establish xenograft tumors, 5×10^6^ 22RV1 or CL1 cells in 150 µl DMEM were subcutaneously injected into the right flank of each mouse. When tumors reached 70–150 mm^3^ in size, mice were randomly assigned to one of four groups (six mice per group). Adenovirus (2×10^9^ PFUs per mouse) or PBS was injected into the tumors every other day, with a total of three injections. The tumor volume (mm^3^) was measured with a vernier caliper every four days and calculated as (length×width^2^)/2. To study viral kinetics *in vivo*, tumors injected with Ad-PTEN or Ad.DD3.D55-PTEN were collected at 3, 6, 9, 12 and 15 days after the last injection and quickly frozen in liquid nitrogen. The total tumor DNA was extracted using the Genomic DNA MiniPreps Kit (Generay Biotech, Shanghai, China). Viral DNA was measured by real-time PCR using primers that anneal to the adenovirus E3 region. The oligonucleotide primers used for amplification are listed in Table 1. Genomic DNA amplified by β-actin primers was used as an internal control.

**Table 1:** Nucleotide sequence of oligonucleotide primers used for virus identification, RT-PCR and real-time PCR.

Gene	Direction	Nucleotide sequence
adenovirus E1A	forward	5′-gaggagtttgtgttagattagattatgtg-3′
	reverse	5′-cagatgagccacataataataagg-3′
GAPDH	forward	5′-ggtgaaggtcggagtcaacgga-3′
	reverse	5′-gagggatctcgctcctggaaga-3′
PTEN	forward	5′-gacgaactggtgtaatgata-3′
	reverse	5′-gtgccactggtctataatcc-3′
adenovirus E3	forward	5′-aacagagatgaccaacacaac-3′
	reverse	5′-gtgccactggtctataatcc-3′
β-actin	forward	5′-tcacccacactgtgcccatctacga-3′
	reverse	5′-cagcggaaccgctcattgccaatgg-3′

### Cells and Cell Culture

The human cell lines used in this study were LNCaP, DU145, 22RV1,PC3 (prostate cancer), T-24 (bladder cancer), BEL-7404 (liver cancer), HeLa (cervical cancer), BEAS-2B (bronchial epithelial cell), HFL-I (embryonic lung fibroblast), L-02 (liver cell) and WISH (amnion cell). All cell lines were purchased from the Cell Bank of the Type Culture Collection of the Chinese Academy of Sciences (Shanghai, China). CL1 cells (prostate cancer) were grown as described by Tso et al. [54]. HEK293 was obtained from Microbix Biosystems, Inc. (Toronto, Ontario, Canada). The cell lines were incubated in DMEM or RPMI 1640 supplemented with 5–10% heat-inactivated fetal bovine serum (FBS) at 37°C in a humidified air atmosphere with 5% CO_2_.

### Luciferase Assay and Construction of Different Adenoviruses

The minimal *DD3* promoter (AF279290 nt309–522) was cloned as described previously [17]. The luciferase reporter plasmids pGL3-basic and pGL3-control were purchased from Promega Corp. (Madison, WI, USA). The woodchuck hepatitis virus post-transcriptional regulatory element (WPRE) was a kind gift from Dr. Xiaoming Xie and was inserted downstream of the luciferase reporter gene between the *Xba*I and *Nhe*I sites in the plasmid pGL3-DD3 to construct the plasmid pGL3-DD3-WPRE. The luciferase reporter gene assay was performed as previously described [17]. All results were the average of three independent experiments.

Different adenoviruses were generated by homologous recombination using Effectene Transfection Reagent (Qiagen, Germany) with the plasmid pBHGE3 and the plasmids pAd.DD3.D55 or pAd.DD3.D55-PTEN. Wild-type adenovirus (Ad.WT) was previously preserved in our laboratory [55]. The viral structure was confirmed by PCR. The sequences of the primers used for PCR are listed in Table 1.

### Western Blot Analysis

The different cells were harvested at various times after infection with the indicated viruses at an MOI of 10. The total protein was extracted in cell lysis buffer (Beyotime, Shanghai, China). Protein concentrations were measured with the Enhanced BCA Protein Assay Kit (Beyotime, Shanghai, China). The protein samples were electrophoretically separated on an 8–12% SDS-polyacrylamide gel and transferred to a PVDF membrane. The membranes were blocked for 30 min with 5% nonfat milk, sequentially incubated with primary and secondary antibodies and finally developed with ECL Western blot detection reagents (Pierce Biotechnology, Rockford, Illinois, USA) according to the manufacturer's instructions. Primary antibodies for E1A, E1B-55K, procaspase-3, poly(ADP-ribose) polymerase (PARP), Akt(Pan)(C67E7) and phospho-Akt(Ser473)(D9E) and all of the secondary antibodies were obtained from Santa Cruz Biotechnology (Santa Cruz, CA, USA). PTEN, cleaved-PARP and procaspase-8 antibodies were purchased from Cell Signaling Inc. (Danvers, MA, USA). The procaspase-9 antibody was purchased from Thermo Fisher Scientific Inc. (Fremont, CA, USA). The XIAP antibody was purchased from R&D Systems Inc. (Minneapolis, MN, USA) and the tubulin antibody was manufactured by Sigma-Aldrich (St. Louis, MO).

### Crystal Violet Cytopathic Assay

Cells were seeded in 24-well plates and infected with Ad-PTEN, Ad.DD3.D55, Ad.DD3.D55-PTEN or wild type adenovirus at various MOIs. Five days after infection, cells were exposed to 2% crystal violet dissolved in 20% methanol for 5 min. The plates were then washed with distilled water (dH_2_O) and documented by photography.

### Colorimetric MTT (Tetrazolium) Cell Viability Assay

Cells cultured in 96-well plates were incubated with viruses at an MOI of 10. MTT solution (20 µl, 5 mg/ml) was added to each well after various times and the absorbance was measured in dual wavelength mode (595 nm and 655 nm). The percentage of cell viability was calculated as follows: (mean A595/A655 of infected cells)/(mean A595/A655 of uninfected cells)×100.

### Apoptotic Cell Staining

Cells grown on glass coverslips were treated with viruses at an MOI of 10 for 48 h and stained with 1 µg/ml Hoechst 33258 (Molecular Probes, Eugene, OR, USA).

### Immunohistochemical Analysis and TdT–mediated dUTP Nick End Labeling (TUNEL) Assay

Frozen tumor sections were incubated in 1% H_2_O_2_ and 0.4% Triton X-100 to eliminate endogenous peroxidase activity. The sections were then blocked with the blocking serum and incubated with the corresponding antibody. The subsequent procedures were performed using the ABC Staining System (Westang, Shanghai, China) according to the manufacturer's instructions.

The apoptotic cells in tumor tissue sections were observed by using terminal deoxynucleotidyl transferase-mediated dUTP-biotin nick end labeling staining with a TACSTM 2 TdT-DAB In Situ Apoptosis Detection Kit (Trevigen, Gaithersburg, MD, USA) according to the manufacturer's instructions. The sections were counterstained with hematoxylin. All sections were visualized with an Olympus BX51 microscope (Olympus, Tokyo, Japan).

### Statistical Analysis

The differences between the groups were assessed using the Student's *t*-test.
